# Methylmercury sorption to polyethylene terephthalate (PET) fibers and relevance to environmental exposure

**DOI:** 10.1093/etojnl/vgae067

**Published:** 2025-01-06

**Authors:** Tom Sizmur, Harrison Frost, Monica Felipe-Sotelo, Tom Bond, Mark L Mallory, Nelson J O’Driscoll

**Affiliations:** Department of Geography and Environmental Science, University of Reading, Reading, United Kingdom; Earth and Environmental Science Department, Acadia University, Wolfville, NS, Canada; School of Civil Engineering & Surveying, University of Portsmouth, Portsmouth, United Kingdom; Department of Chemistry, University of Surrey, Guildford, United Kingdom; Department of Chemistry, University of Surrey, Guildford, United Kingdom; School of Sustainability, Civil and Environmental Engineering, University of Surrey, Guildford, United Kingdom; Biology Department, Acadia University, Wolfville, NS, Canada; Earth and Environmental Science Department, Acadia University, Wolfville, NS, Canada

**Keywords:** methylmercury, microplastic, adsorption, polyethylene terephthalate, bioaccumulation

## Abstract

Considerable amounts of polyethylene terephthalate (PET) microplastic fibers are released into the environment by the laundering of polyester clothing. Microplastic fibers can be ingested by organisms in the environment. Therefore, it has been suggested that microplastic fibers act as vectors for adsorbed contaminants, which are subsequently desorbed in the gut of the organism. We undertook sorption isotherm experiments at pH 6, 7, and 8 to quantify the sorption of methylmercury (MeHg) to PET fibers. Sorption isotherms were fit to Langmuir, Freundlich, and Brunauer–Emmett–Teller models. Sorption decreased with increasing pH, which can be explained by physisorption on the negatively charged PET surfaces and the greater presence of neutral or negatively charged MeHg species at higher pH. We used the parameters obtained by the model fits to predict the likely concentration of MeHg on PET microplastic fibers in aquatic ecosystems with environmentally realistic MeHg concentrations. We calculated MeHg concentrations on PET microplastic fibers to be four orders of magnitude lower than previously observed concentrations of MeHg in seston (suspended particles comprising algae and bacteria) at the base of the aquatic food web. The results indicate that the presence of PET microplastic fibers in the environment do not elevate the MeHg exposure to organisms that ingest fibers in the environment.

## Introduction

Over 350 million tonnes of plastics are produced annually, resulting in more than 240 million tonnes of plastic waste (Joseph et al., 2024). Polyethylene terephthalate (PET) is one of the most commonly manufactured plastics used to make synthetic fabrics and food industry packaging, resulting in widespread contamination of air, soil, sediment, groundwater, and oceans (Dhaka et al., 2022; Dris et al., 2017). When PET clothing is laundered, it sheds thousands of fibers (Frost et al., 2020) that enter wastewater treatment plants, where it can then partition into either the wastewater or, more often, the sewage sludge (Frost et al., 2022). In many countries, the application of sewage sludge to land as biosolids represents the primary means of disposal (Collivignarelli et al., 2019). Microplastics in soils have the potential to cause harm to soil biodiversity, food safety, and human health (Wang et al., 2019). Furthermore, point source pollution from wastewater discharges and diffuse leaching from agricultural land to which biosolids have been applied both represent sources of microplastics to rivers (Kay et al., 2018; Schell et al., 2021) and, ultimately, sediments in the freshwater and marine aquatic environment (Leslie et al., 2017). Both aquatic and terrestrial organisms ingest microplastics (Bertoli et al., 2022; Lahive et al., 2022; Vecchi et al., 2021), perhaps mistaking them for natural organic matter (Egbeocha et al., 2018). It is therefore possible that PET fibers may be a vector for toxic chemicals to organisms if sorption of toxic chemicals occurs prior to ingestion (Frost et al., 2022).

The concentrations of mercury (Hg) in soils, freshwater, and oceans are elevated due to atmospheric deposition of anthropogenic emissions, principally from fossil fuel combustion and artisanal and small-scale gold mining (Pacyna et al., 2016). Inorganic Hg(II) can be biologically methylated to generate methylmercury (MeHg) by microorganisms possessing *hgcAB* genes inhabiting a wide range of microbial habitats (Paranjape & Hall, 2017; Tang et al., 2020). The percentage of Hg present as MeHg ranges from 0.001% to 21.25% in sediments (Dai et al., 2021) and has been observed to be up to 50% in ocean waters (Bowman et al., 2020). Methylmercury is more toxic and biomagnifies up trophic levels in food webs (Munthe et al., 2019). Elevated concentrations at high trophic levels can result in neurological disorders in wildlife (Chételat et al., 2020) or human populations (Kim et al., 2016). The bioconcentration of MeHg from the water column into seston (suspended particles comprising algae and bacteria) is the most important factor predicting MeHg concentrations in organisms at higher trophic levels (Wu et al., 2019). Organisms at the base of food webs, such as benthic filter feeders (Fabra et al., 2021; Porter et al., 2023), zooplankton (Geng et al., 2021; Ogonowski et al., 2016), or juvenile fish (Ory et al., 2018), ingest microplastics. Organisms at higher trophic levels can thus also be exposed by ingestion of microplastic-contaminated prey through trophic transfer, consuming organisms that have already ingested microplastics (Athey et al., 2020; Provencher et al., 2019). The transfer of microplastics may occur from a prey species with microplastics retained in their gut to a predator, but it is also possible for microplastics to be absorbed through the gut and bioaccumulate within the tissues of biota (Browne et al., 2008). Therefore, if microplastic fibers are a vector of MeHg to organisms at the base of food webs (i.e., where concentrations are often below the parts per trillion range), then they may play an important role in the exposure of MeHg to organisms at higher trophic levels where loadings of MeHg can reach levels that have deleterious effects (i.e., where concentrations are often in the parts per million range). Quantifying MeHg adsorption to microplastic fibers is important to judge whether plastic pollution increases the risk of MeHg bioaccumulation at the base of the food web.

While sorption of pollutants to microplastics has been investigated (Brennecke et al., 2016; Gao et al., 2021; Menéndez-Pedriza & Jaumot, 2020), there is little research focusing on PET microplastic fibers, relative to their importance as the most common synthetic fiber used in textiles (Frost et al., 2022; Zhang et al., 2020). Experimental data quantifying MeHg sorption to microplastics is limited in the literature. However, Taylor et al. (2019) compared MeHg sorption to polyethylene, polyoxymethylene, polysulfone, polyethersulfone, and polyphenylene sulfide to identify a suitable plastic to be used in passive samplers and found a high affinity of MeHg to sulfur-containing polysulfone and polyphenylene sulfide. This observation supports a well-characterized binding of MeHg, a soft Lewis acid, with soft Lewis bases such as thiol (sulfhydryl) groups (Skrobonja et al., 2019). The chemical structure of PET is such that alternating units of terephthalate groups and ethylene groups are terminated with a hydroxyl group. Methylmercury can be present as a negatively, neutral, or positively charged ion in solution, depending on pH (Blanc et al., 2018). Therefore, while the surface of PET may be electronegative (Ortega & Cortés-Arriagada, 2023), favoring electrostatic physisorption of positively charged MeHg ions through cation–π interactions in lower pH solutions, the presence of hydroxyl groups at the ends of polymer chains may allow pH-dependent chemisorption due to deprotonation of the hydroxyl groups in higher pH solutions. Methylmercury can also exhibit hydrophobic properties and adsorb to surfaces as a hydrophobic organic micropollutant (Turner & Millward, 2002).

We undertook laboratory experiments to quantify the sorption of MeHg to PET fibers in aqueous solutions buffered to pH 6, 7, and 8. Our objective was to characterize the shape of the sorption isotherm across a range of MeHg concentrations to gain insights regarding the sorption mechanisms and to predict the likely microplastic fiber MeHg loading in realistic environmental media at the base of the aquatic food web. We hypothesized that sorption would be pH-dependent with greater sorption occurring at higher pH due to chemisorption. We also hypothesized that sorption of MeHg to PET in solutions containing realistic environmental MeHg concentrations would result in greater MeHg loadings than naturally occurring seston (suspended particles comprising algae and bacteria).

## Methodology

### Materials

The PET fibers used for this experiment were generated by grinding PET fabric in a cryogenic mill to produce fibers with an average length of 174.1 ± 131.8 µm and a point of zero net charge of 1.95 (Frost et al., 2024). Characterization by optical microscopy and Raman spectroscopy of the fibers revealed that cryo-milled fibers were representative of those produced during laundering, and that the milling process did not cause significant chemical alteration of the materials (Frost et al., 2024).

### Sorption isotherms

Sorption isotherms were conducted by shaking 5 ± 0.1 mg of microplastic fibers on a reciprocal shaker at 200 movements min^−1^ for 6 hr at 20°C in a polypropylene microcentrifuge tube with 1 ml of 0.01 mol L^−1^ phosphate buffer (pH 6, 7, or 8) containing MeHg (5, 25, 50, 100, 200, 300, or 500 µg L^−1^), resulting in a solid:liquid ratio of approximately 1:200. These concentrations were not selected to represent realistic environmental concentrations, but to generate sorption isotherms, which could be modeled to infer the likely sorption mechanisms and predict the sorption capacity of the microplastic fibers. Buffered solutions of MeHg were made by dilution of a certified 2 mg L^−1^ MeHg stock solution (Brooks Rand) and a defined ratio of monobasic sodium phosphate and dibasic sodium phosphate (See online supplementary material, Table SI-1). Four replicates of each combination of pH and MeHg concentration were prepared, resulting in a total of 84 samples.

After shaking, solutions were centrifuged at 5,000 rpm for 1 min, a 100 µl aliquot was passed through a filter pipette tip, diluted, acidified with 0.5% HCl, and refrigerated prior to analysis the following day. An aliquot of the filtered sample was added to a 40 ml glass vial along with 2 ml of 2 mol L^−1^ acetate buffer and topped up with ultrapure water (>18.2 MΩ). A different dilution factor and aliquot volume was used for each sample depending on the MeHg concentrations in the solutions added to the microplastic fibers (See online supplementary material, Table SI-2) to bring all the samples into a similar range for analysis (100–200 pg). Immediately before a septa cap was screwed on the top of the vial, 50 µl of a 1% sodium tetraethyl borate (C_8_H_20_BNa) solution in 2% KOH was added to the vial to ethylate the Hg species prior to chromatographic separation, following Bloom (1989).

### MeHg analysis

Vials were inverted to mix prior to analysis following the US Environmental Protection Agency method 1630 (US Environmental Protection Agency, 1998) ethylation purge and trap approach on a Brooks Rand MERX Model III Gas Chromatography Atomic Fluorescence Spectroscopy (Mansfield & Black, 2015). The instrument was calibrated using solutions prepared with the same protocol as the samples but, instead of the sample, a solution containing 1, 10, 50, 100, 200, or 500 pg of MeHg was added. These calibration standards were made by adding appropriate aliquots of 0.01 µg L^−1^ or 1 µg L^−1^ MeHg solutions made by serial dilution of a certified 1 mg L^−1^ MeHg stock (Brooks Rand). Samples were analyzed alongside method blanks containing pH 6, 7, and 8 phosphate buffers without standard or sample addition. A 50 pg MeHg check standard was analyzed in triplicate at the start and end of the run and every 20 samples to check for instrument drift, for which an average recovery of 97% (SD = 7%, *n* = 27) was obtained. Spiked recoveries were created by adding 50 pg MeHg to individual samples with an average recovery of 100% (SD = 10%, *n* = 8).

### Sorption model fitting

The quantity of MeHg sorbed to the microplastic fibers was calculated using the following equation:


$$Cs=\frac{\left(Ci-\mathrm{Caq}\right)\times V}{Sm}$$

where *Cs* is the MeHg concentration on the microplastic fibers (µg g^−1^), *Ci* is the initial solution MeHg concentration (µg L^−1^), a nominal value reported in online supplementary material Table SI-3, *Caq* is the final solution MeHg concentration (µg L^−1^), *V* is the solution volume (L), and *Sm* is the mass of microplastic fibers weighed out (g). Raw average *Cs* and *Caq* values are provided in online supplementary material Table SI-3.

We then fit the sorption isotherms to three different models, following Desauziers et al. (1997), to examine the pattern of sorption of MeHg with microplastics. First, the sorption data were fit to the Langmuir model using the following equation:


$$\frac{Cs}{\mathrm{Caq}\;}=\frac{b\;\mathrm{Csm}}{1+\mathrm{Caq}\;b}$$

where *Cs* is as defined above, *Caq* is the final solution MeHg concentration (µg L^−1^), *b* is the Langmuir binding constant, and *Csm* is the maximum sorption capacity of MeHg on the microplastic fibers (µg g^−1^). The Langmuir model assumes that adsorption occurs as a monolayer across specific adsorption sites with one homogenous adsorption energy and no interactions between adsorbates (Desauziers et al., 1997).

We also fit the sorption data to the Freundlich model using the following equation:


Cs=Kf Caqn1

where *Cs* is as defined above, *Caq* is the final solution MeHg concentration (µg L^−1^), *Kf* is the Freundlich sorption capacity parameter, and *n* is the Freundlich intensity parameter. The Freundlich model assumes adsorption across heterogenous binding sites with different adsorption energies (Desauziers et al., 1997).

Finally, we fit the sorption data to the Brunauer–Emmett–Teller (BET) model using the following equation:


$$Cs=\;\frac{Q{mK}_{1}Caq}{\left(1+\;K_{1}Caq+\frac{\mathrm{Caq}}{Cm}\right)\;\left(1-\frac{\mathrm{Caq}}{Cm}\right)}$$

where *Cs* is as defined above, *Caq* is the final solution MeHg concentration (µg L^−1^), *K_1_* is the isotherm constant, *Q* is quantity of MeHg sorbed on the microplastic fibers when the monolayer is saturated (µg g^−1^), and *Cm* is the MeHg concentration (µg L^−1^) in solution when the microplastic fibers are saturated with respect to MeHg. The BET model assumes multilayer adsorption (Desauziers et al., 1997).

### Comparisons between microplastic fibers and seston in realistic environments

Wu et al. (2019) conducted a meta-analysis of studies reporting MeHg concentrations in aqueous solutions and in seston (suspended particles comprising algae and bacteria) at the base of the aquatic food web. We extracted data relating to each aquatic ecosystem where Wu et al. (2019) reported MeHg concentrations in both seston and water (at pH between 5.5 and 8.5). We used the MeHg concentration in water as a *Caq* value in our parameterized Langmuir, Freundlich, and BET models to predict the MeHg concentration that would occur on the PET microplastic fibers (i.e., the *Cs* value) at each aquatic ecosystem site. Because we derived Langmuir, Freundlich, and BET model parameters, which relate to pH 6, 7, and 8, we used the most appropriate model parameters for the pH of the water at the sites, as reported by Wu et al. (2019). Our pH 6, 7 and 8 Langmuir, Freundlich, and BET model parameters were used to predict *Cs* at Wu et al. (2019) sites with pH 5.5–6.5, 6.5–7.5, and 7.7–8.5, respectively. Predicted concentrations of MeHg adsorbed to PET microplastic fibers (*Cs*) were then compared with the concentration of MeHg associated with seston observed by Wu et al. (2019).

## Results

### MeHg sorption to microplastic fibers

**Figure 1.**
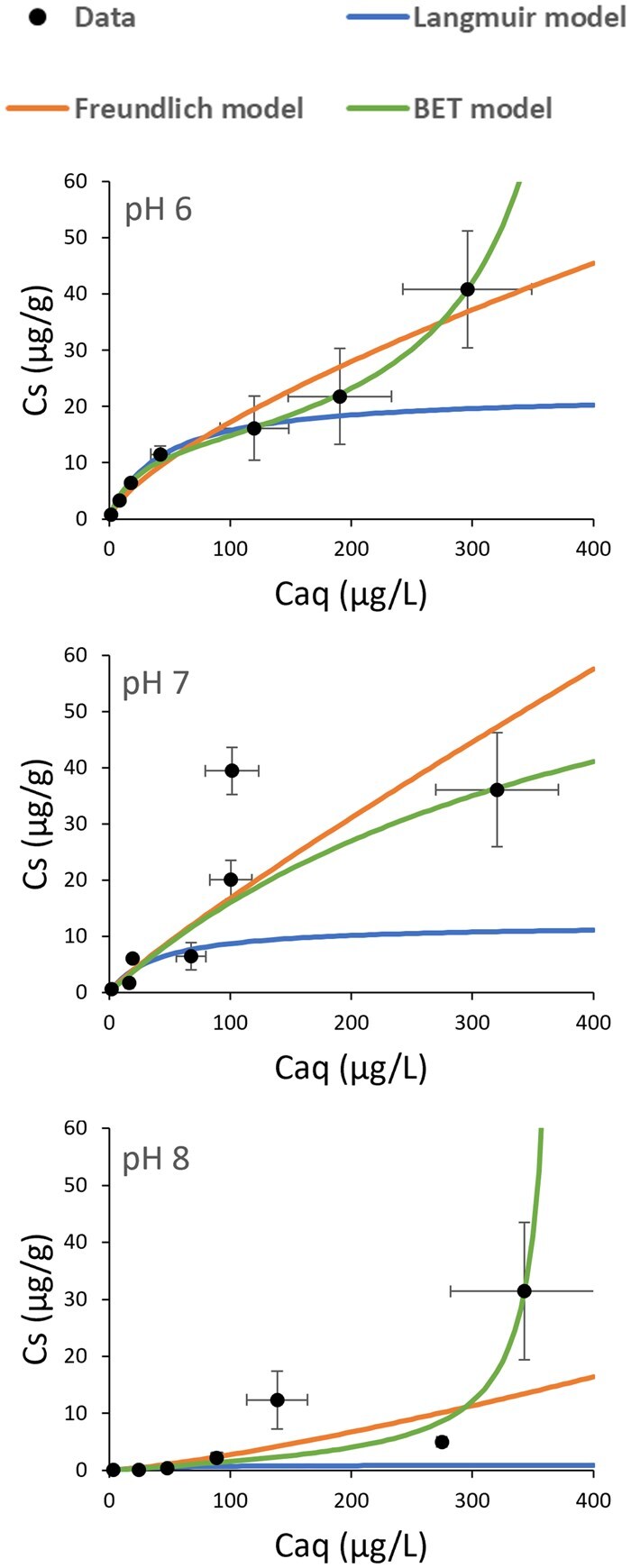
Sorption isotherms for methylmercury (MeHg) sorption to polyethylene terephthalate (PET) microplastic fibers at pH 6, 7, and 8 where *Cs*, the MeHg concentration on the microplastic fibers (µg g^−1^), is plotted against *Caq*, the final solution MeHg concentration (µg L^−1^), and fit to a Langmuir model, Freundlich model, and Brunauer–Emmett–Teller (BET) model. Error bars are standard errors of the mean (*n* = 4).

The maximum MeHg sorption observed was 40.9 µg g^−1^ in a solution containing 500 µg L^−1^ MeHg buffered at pH 6. Solutions containing the same concentration of MeHg resulted in 36.1 and 31.4 µg g^−1^ sorption by the microplastic fibers at pH 7 and 8, respectively. These results indicate an apparent increase in sorption with decreasing pH (Figure 1) and this is supported by a greater Langmuir maximum sorption capacity parameter (*Csm*) and a greater Freundlich sorption capacity parameter (*Kf*) with decreasing pH (Table 1). Similarly, in solutions containing 5 µg L^−1^ MeHg, the lowest concentration evaluated, sorption was higher at pH 6 (0.723 µg g^−1^) than at pH 7 (0.597 µg g^−1^), or pH 8 (0.084 µg g^−1^). However, the parameter representing quantity of MeHg sorbed on the microplastic fibers when the monolayer is saturated (*Q*), derived from the fit of the data to the BET model was higher in pH 7 solutions (85.8 µg g^−1^) than pH 8 solutions (50.0 µg g^−1^), or pH 6 solutions (14.5 µg g^−1^).

**Table 1.** Constants and correlation coefficients for fits of sorption isotherm data for methylmercury (MeHg) sorption to polyethylene terephthalate (PET) microplastic fibers at pH 6, 7, and 8 to the Langmuir Model, Freundlich Model, and Brunauer–Emmett–Teller (BET) model.

pH	6	7	8
Langmuir model			
*Csm*	22.4	12.2	0.992
B	0.0235	0.0249	0.0263
*R*^2^	0.998	0.944	0.814
Freundlich model			
*Kf*	0.682	0.282	0.00749
N	1.43	1.13	0.779
*R*^2^	0.980	0.874	0.830
BET model			
Q	14.5	85.8	50.0
K_1_	0.0457	0.00230	0.000301
Cm	423	232000	373
*R*^2^	0.998	0.631	0.856

*Note*. All data are provided to three significant figures. BET = Brunauer–Emmett–Teller; *Csm* = maximum sorption capacity parameter.

### Goodness of sorption isotherm model fits

There was an overall high degree of within-treatment variation in the dataset, with overall average relative standard deviation values of 35%, 40%, and 69% for the pH 6, 7, and 8 buffered sorption isotherms, respectively. This may be caused by the heterogeneity of the PET fibers between experimental units, but unlikely to be caused by organic growth on the fibers since this was not observed. Nevertheless, the sorption models generally explained the majority of the variation in the data (Table 1). The fits to the Langmuir model resulted in *R*^2^ values of 0.998, 0.944, and 0.814 for the pH 6, 7, and 8 buffered experiments, respectively. The fits to the Freundlich model resulted in *R*^2^ values of 0.980, 0.874, and 0.830, and the fits to the BET model resulted in *R*^2^ values of 0.998, 0.631, and 0.856, respectively. No outliers (treatments or individual replicates) were identified or removed from the dataset. However, there were individual treatments where all four replicates displayed sorption that deviated considerably from the Freundlich model. These treatments include solutions with initial concentrations of 300 µg L^−1^ buffered at pH 7 and 8 where uncharacteristically high and low sorption, respectively, was observed. Therefore, based on the *R*^2^ values (Table 1), the pH 6 isotherm generally fit all three models better than the pH 7 or 8 isotherms. The pH 6 isotherm data fit the BET model particularly well (*R*^2^ = 0.998). However, this model did not provide a good fit to the pH 7 isotherm data (*R*^2^ = 0.631) and is probably the reason why an unintuitively high Q parameter was observed for the pH 7 solutions. Overall, the Freundlich model fit the data the best (*R*^2^ values of 0.980, 0.874, and 0.830 for the pH 6, 7, and 8 buffered experiments, respectively).

### Comparisons between microplastic fibers and seston in realistic environments

**Figure 2.**
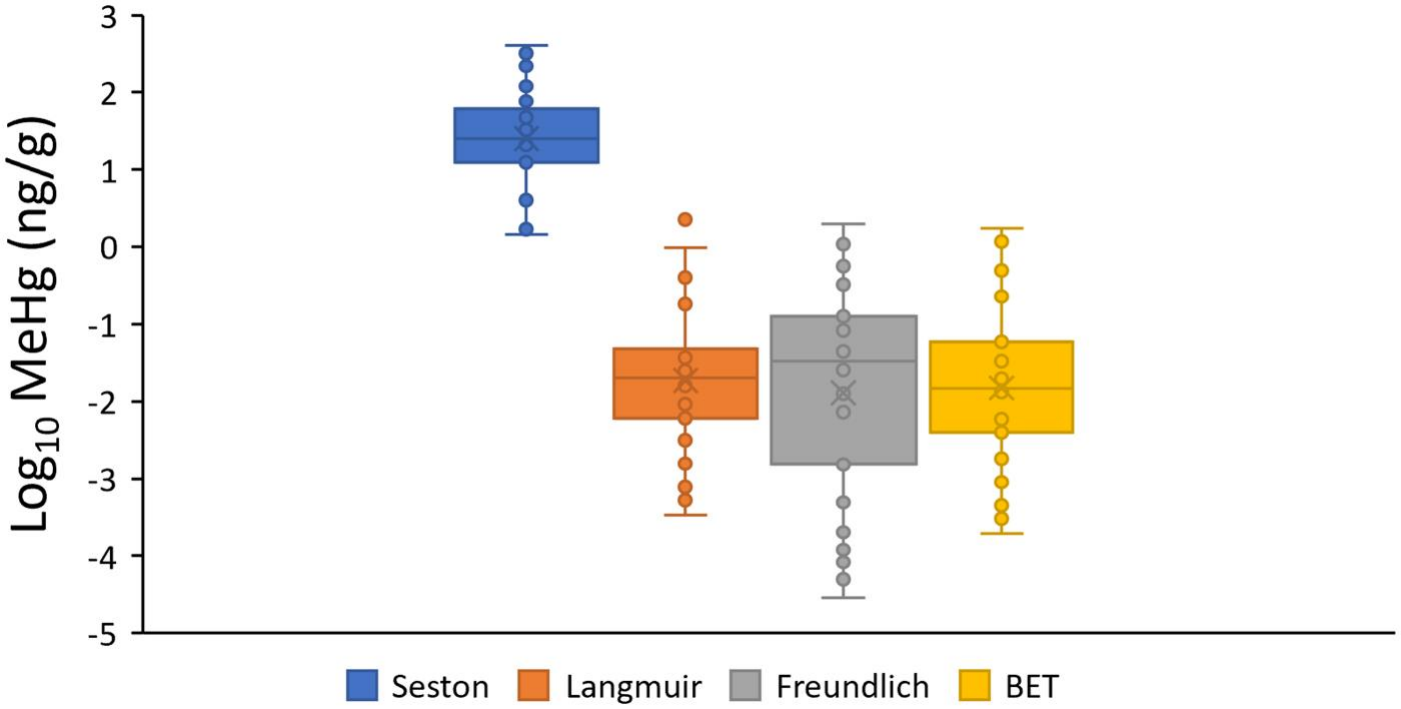
Methylmercury (MeHg) concentrations in seston reported in a global meta-analysis by Wu et al. (2019) alongside predicted concentrations of MeHg sorbed to polyethylene terephthalate (PET) microplastic fibers based on the same concentrations in water, predicted using parameters of the Langmuir, Freundlich, and Brunauer–Emmett–Teller (BET) sorption models.

Regardless of which model parameters were used to predict the hypothetical sorption of MeHg to PET microplastic fibers at realistic environmental concentrations, the median sorption to fibers was more than four orders of magnitude lower than the concentrations of MeHg observed in seston at the aquatic ecosystem sites reported by Wu et al. (2019;  Figure 2). The MeHg concentrations observed at the aquatic ecosystem sites ranged from 0.013 to 9.1 ng L^−1^ and concentrations of MeHg measured in seston ranged between 1.43 and 410 ng g^−1^, with a median concentration of 25.2 ng g^−1^ (See online supplementary material, Table SI-4). By contrast, the concentrations of MeHg predicted to be sorbed on PET microplastic fibers ranged between 0.00003 and 2.51 ng g^−1^, with a median of 0.020, 0.033, or 0.0002, depending on whether the Langmuir, Freundlich, or BET parameters were used to predict the sorption. On average, MeHg loadings on PET microplastic fibers was predicted to be 20,181 times, 191,434 times, or 34,604 times lower (i.e., at least four orders of magnitude lower) than concentrations measured by Wu et al. (2019) in seston, using Langmuir, Freundlich, or BET parameters, respectively.

## Discussion

### Possible sorption mechanisms

Various authors have attributed sorption mechanisms to physisorption (Purwiyanto et al., 2020; Wang et al., 2022) or chemisorption (Tang et al., 2021; Wang et al., 2020), with the relative contribution of each mechanism depending on polymer type, the properties of the adsorbate, and the pH of the solution (Cao et al., 2021). The electronegativity of PET is brought about by two distinct mechanisms. The benzene ring within the terephthalate group contains delocalized electrons and is therefore capable of contributing electrostatic interactions (physisorption) with metal cations in solution (Zhou et al., 2020). The ends of polymer chains are characterized by terminal hydroxyl groups (Patterson & Ward, 1957), which are capable of becoming deprotonated at high pH and undertaking cation exchange with metals in solution (Estrada-Flores et al., 2020). If the pH is above the point of net zero charge (pH_PZC_), changes in solution pH do not alter the electronegativity of the PET that is caused by the delocalized electrons associated with the benzene ring in the terephthalate groups. However, the deprotonation of hydroxyl groups with increasing pH makes the PET more electronegative and raises the chances of chemisorption of cations occurring.

**Figure 3.**
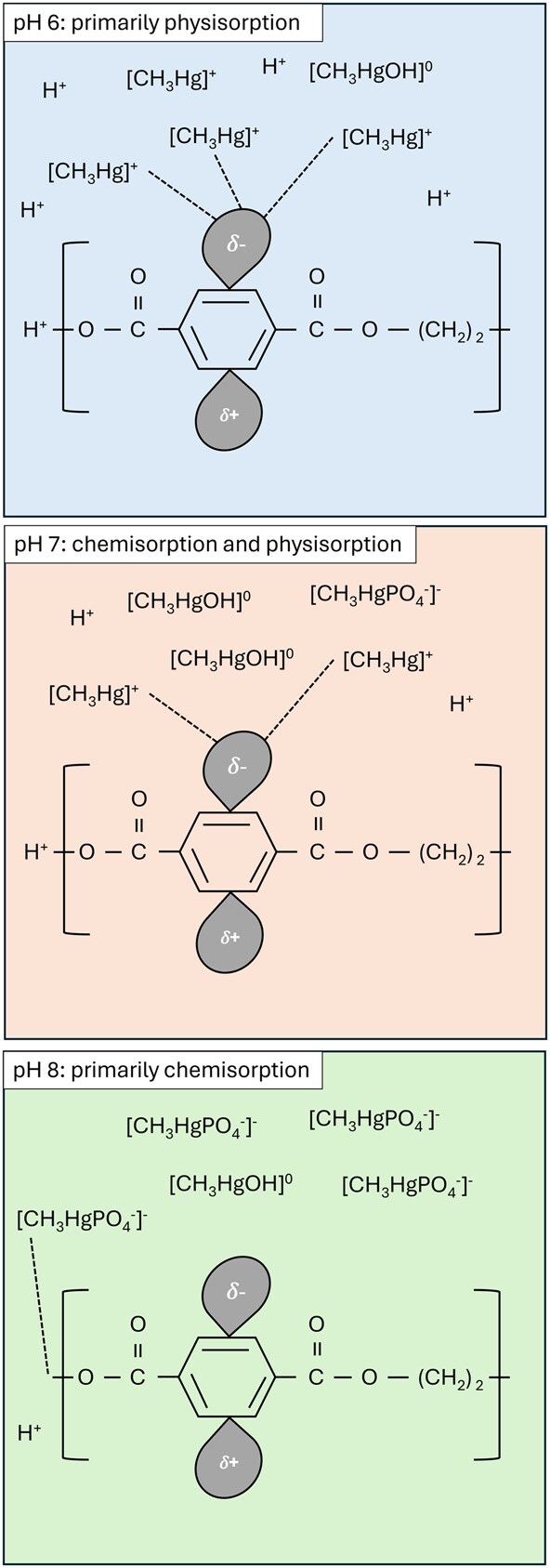
Conceptual model describing proposed mechanisms for methylmercury (MeHg) sorption to polyethylene terephthalate (PET) microplastic fibers at pH 6, 7, and 8.

As pH increases, the MeHg ion is less likely to be in the CH_3_Hg^+^ form and more likely to be present as neutral (e.g., CH_3_HgOH^0^) or negatively (e.g., CH_3_${\text{HgPO}}_{4}^{–}$) charged complexes in phosphate-buffered solutions in the absence of dissolved organic matter or chloride ions (Loux, 2007; Muller et al., 2019). These neutral or negative aqueous complexes do not adsorb to negatively charged surfaces (Figure 3). It therefore seems likely that the primary mechanism for MeHg sorption directly on plastic surfaces, as observed in our experiments, was through electrostatic attraction (physisorption) of negatively charged MeHg ions to the PET surfaces. This tendency toward physisorption probably decreases with increasing pH as the MeHg in solution is less positively charged (Blanc et al., 2018). This interpretation is supported by the Freundlich parameter *n* because *n* > 1 in pH 6 and 7 solutions, indicative of physisorption, and *n* < 1 in pH 8 solutions, which would suggest chemisorption (Purwiyanto et al., 2020).

A good fit with the BET model was observed by Desauziers et al. (1997) when quantifying sorption of MeHg on clay minerals and metal oxides. They proposed a two-step process whereby a monolayer first becomes saturated (either by physisorption or chemisorption) followed by a second phase at higher solution concentrations where hydrophobic interactions occur between methyl groups of MeHg in solution and on the sorbate. Therefore, after initial electrostatic sorption of MeHg to PET surfaces, multilayer sorption may occur due to hydrophobic interactions between the methyl groups on MeHg. However, it is unlikely that sufficient concentrations of MeHg occur in the natural environment to result in a complete monolayer coverage on the surfaces of microplastics.

### MeHg associated with microplastics several orders of magnitude lower than naturally occurring seston

The premise of the argument that microplastics can act as vectors for the bioaccumulation of contaminants by organisms is that microplastics are able to adsorb contaminants from solution (Frost et al., 2022; Tumwesigye et al., 2023). This premise has led to some papers reporting that microplastics increase the uptake and toxicity of contaminants to organisms (Rainieri et al., 2018), others reporting a decrease (Oliveira et al., 2018), and others reporting no clear effect (Fernández et al., 2020). Often experimental conditions vary between experiments and differ from real contaminated environments. However, a key reason for contrasting conclusions about the role of microplastics in the bioaccumulation of contaminants is that they all test different hypotheses (Koelmans et al., 2016). Many experiments simply compare solutions containing contaminants in aqueous solution to the same solutions containing contaminants and microplastics (e.g., Barboza et al., 2018; Rainieri et al., 2018). However, perhaps a more appropriate and environmentally relevant comparison is to compare the dose of contaminants organisms receive when ingesting contaminated microplastics to the dose received when ingesting other naturally occurring particles in water bodies.

We calculated that MeHg concentrations on PET microplastic fibers would be approximately four orders of magnitude lower than the concentrations observed in seston (suspended particles comprising algae and bacteria) across a globally distributed range of sites collated by Wu et al. (2019). We therefore conclude that the accidental ingestion of PET microplastic fibers by invertebrates foraging for seston is unlikely to directly increase the bioaccumulation of MeHg by the foraging organisms, based on our data. Because MeHg sorption to PET fibers was so much lower than concentrations in naturally occurring seston, it is also unlikely that microplastics in aqueous systems will scavenge MeHg and reduce the accumulation of MeHg on seston particles. Furthermore, microplastics are more likely to be eliminated from the tissues of ingesting organisms and not digested, because they are not nutritive particles, resulting in greater elimination rates of associated contaminants (Rivera-Hernández et al., 2019). Seston include particles that are highly colonized with bacteria or algae and so there is already a bioconcentration factor from the water column to these single-celled organisms that constitutes a crucial step in the MeHg biomagnification pathway (Gojkovic et al., 2023).

### Future work

The microplastic fibers used in this study were pristine in the sense that they had not been artificially aged or allowed to be colonized by microorganisms. Several studies have demonstrated that the sorption of Hg to microplastics is greater after the plastics have been aged by exposure to UV irradiation (Pinto et al., 2022) or oxidizing agents such as H_2_O_2_ (Gao et al., 2023). Microplastics in the natural environment become colonized by a community of bacteria and algae that is distinct from communities that develop on natural particles (Miao et al., 2019). Further work could investigate whether these communities result in microplastic fibers containing higher concentrations of MeHg due to bioaccumulation. Furthermore, the methylation of inorganic Hg may be accelerated by the presence of microplastics due to the colonization of Hg-methylating bacteria on the plastic surfaces (Bowman et al., 2021; Hao et al., 2023).

The MeHg concentrations used in this study (5–500 µg L^−1^) were much higher than environmentally observed concentrations, which are typically within the ng L^−1^ range (Mousavi et al., 2011). The concentrations were selected for the purposes of fitting sorption isotherm models to the data to elucidate sorption mechanisms. They are not intended to reflect typical maximum sorption capacities in real environments. Sanders et al. (2020) undertook MeHgOH sorption isotherm experiments using L-cysteine-functionalized PET at the more realistic environmental concentration of 0.05 µg L^−1^ and observed sorption in the range 0.98–1.56 ng g^−1^. This is approximately two orders of magnitude greater than our fits to the Wu et al. (2019) data (mostly likely due to the strong bond between MeHg and the thiol group on the cysteine molecule) but still an order of magnitude lower than the concentrations observed in seston. Likewise, deployment of sulfur-containing polysulfonate and polyphenylene sulfide polymers in estuarine sediment mesocosms by Taylor et al. (2019) resulted in concentrations of 6.37 and 0.72 ng g^−1^, respectively. But these were an order of magnitude lower than the concentrations they measured in amphipods (39.6 ng g^−1^) or sediments (13.6 ng g^−1^).

The chemical composition and physical conditions of the solutions used in our experiments do not reflect natural waters in terms of temperature, ionic strength, and dissolved organic species. Of these factors, the presence of dissolved organic matter plays a key role not only in the methylation of Hg (Wu et al., 2022), but also generally reduces the sorption of MeHg to the surfaces of engineered materials by binding with MeHg in solution (Muller et al., 2019). Therefore, due to the likely sorption of MeHg to plastic tubes and filters in our experiment, and blocking/fouling of sorption sites on the surface of microplastic fibers by naturally occurring dissolved organic matter, our sorption isotherms likely overestimate the MeHg sorption to microplastics in the natural environment.

## Conclusions

Methylmercury sorption to PET microplastic fibers decreased with increasing pH, most likely due to the presence of neutral or negatively charged MeHg species in solution. The results indicated that the primary mechanism for MeHg sorption to PET microplastic fibers was physisorption due to the delocalized electrons of the benzene ring in the terephthalate groups when the pH is above the point of net zero charge (pH_PZC_). The concentration of MeHg on PET microplastic fibers in solutions containing MeHg at environmental concentrations was calculated to be four orders of magnitude lower than the concentration of MeHg observed in seston (suspended particles comprising algae and bacteria) across a globally distributed range of aquatic ecosystem sites reported by Wu et al. (2019). Our findings indicate that the presence of PET microplastic fibers in the environment do not elevate the exposure of MeHg to organisms that ingest microplastic fibers.

## Supplementary Material

vgae067_Supplementary_Data

## Data Availability

All raw data collected during the preparation of the manuscript are provided in the Supplementary Data file.
