# Supplementary material for: Methylmercury sorption to polyethylene terephthalate (PET) fibers and relevance to environmental exposure
Source: Environ Toxicol Chem. 2025 Jan 6;44(2):335–43. doi: 10.1093/etojnl/vgae067 (PMC11816310; doi:10.1093/etojnl/vgae067)
Supplement: vgae067_Supplementary_Data [file vgae067_supplementary_data.docx]

Supplementary Data for:

**Methylmercury sorption to polyethylene terephthalate (PET) fibres and relevance to environmental exposure**

Tom Sizmur^1,2^, Harrison Frost^3,4^, Monica Felipe-Sotelo^4^, Tom Bond^5^, Mark L. Mallory^6^, and Nelson J. O’Driscoll^2^

^1^Department of Geography and Environmental Science, University of Reading, Reading, UK

^2^Earth and Environmental Science Department, Acadia University, Wolfville, Nova Scotia, Canada

^3^School of Civil Engineering & Surveying, University of Portsmouth, Portsmouth, UK

^4^Department of Chemistry, University of Surrey, Guildford, UK

^5^School of Sustainability, Civil and Environmental Engineering, University of Surrey, Guildford, UK

^6^Biology Department, Acadia University, Wolfville, Nova Scotia, Canada

This Supplementary Data file contains four tables (Table SI-1, Table SI-2, Table SI-3, and Table SI-4) cited in the manuscript

**Table SI-1 concentrations of monobasic sodium phosphate and dibasic sodium phosphate mixed to create 0.01 mol L^-1^ phosphate buffers**

| pH | Monobasic sodium phosphate (NaH_2_PO_4_⋅H_2_O) concentration (mol L^-1^) | Dibasic sodium phosphate (Na_2_HPO_4_⋅2H_2_O) concentration (mol L^-1^) |
| --- | --- | --- |
| 6 | 0.0088 | 0.0012 |
| 7 | 0.00423 | 0.00577 |
| 8 | 0.09384 | 0.00932 |

**Table SI-2 dilution factors and volumes of samples added to 40 ml glass vials for MeHg analysis**

| Initial solution MeHg concentration (µg L^-1^) | Dilution factor | Vol added to 40 ml glass vial for analysis (µl) |
| --- | --- | --- |
| 5 | 20 | 800 |
| 25 | 100 | 800 |
| 50 | 100 | 400 |
| 100 | 100 | 200 |
| 200 | 200 | 200 |
| 300 | 200 | 150 |
| 500 | 200 | 80 |

**Table SI-3 *Cs* (MeHg concentration on the microplastic fibres) and *Caq* (the final solution MeHg concentration) values for each *Ci* (initial solution MeHg concentration) and pH**

|  | pH 6 | | pH 7 | | pH 8 | |
| --- | --- | --- | --- | --- | --- | --- |
| Ci (µg L^-1^) | Cs (µg g^-1^) | Caq (µg L^-1^) | Cs (µg g^-1^) | Caq (µg L^-1^) | Cs (µg g^-1^) | Caq (µg L^-1^) |
| 5 | 0.72 | 1.41 | 0.60 | 2.04 | 0.08 | 3.33 |
| 25 | 3.28 | 8.51 | 1.69 | 16.48 | 0.14 | 24.27 |
| 50 | 6.41 | 17.92 | 6.11 | 19.43 | 0.45 | 47.76 |
| 100 | 11.49 | 42.13 | 6.43 | 67.57 | 2.20 | 88.89 |
| 200 | 16.13 | 119.86 | 20.06 | 100.46 | 12.37 | 138.77 |
| 300 | 21.81 | 190.53 | 39.46 | 101.38 | 5.02 | 274.71 |
| 500 | 40.90 | 295.94 | 36.10 | 320.32 | 31.44 | 342.94 |

**Table SI-4 Concentrations of MeHg adsorbed on PET microplastic fibres at sites with known MeHg concentrations in waters predicted using parameters derived from Langmuir, Freundlich, and BET model fits to sorption isotherm data, presented alongside concentrations analysed in seston at each site.**

| Site* | pH* | Water*  ng L^-1^ | Seston*  ng g^-1^ | Langmuir  ng g^-1^ | Freundlich  ng g^-1^ | BET  ng g^-1^ |
| --- | --- | --- | --- | --- | --- | --- |
| 7.13 | 5.60 | 0.77 | 51.1 | 0.39877 | 0.56824 | 0.49255 |
| 7.02 | 5.64 | 0.09 | 94.2 | 0.04735 | 0.12619 | 0.05935 |
| 7.03 | 5.88 | 0.02 | 32.5 | 0.01054 | 0.04397 | 0.01323 |
| 16 | 6.00 | 1.94 | 51.6 | 0.97825 | 1.08605 | 1.18038 |
| 13 | 6.07 | 0.03 | 5.0 | 0.01581 | 0.05842 | 0.01984 |
| 3.02 | 6.10 | 0.05 | 40.0 | 0.02633 | 0.08358 | 0.03303 |
| 7.09 | 6.17 | 0.07 | 16.6 | 0.03685 | 0.10581 | 0.04620 |
| 7.11 | 6.18 | 0.35 | 21.2 | 0.18303 | 0.32696 | 0.22811 |
| 12 | 6.25 | 0.45 | 61.2 | 0.23478 | 0.38994 | 0.29197 |
| 7.12 | 6.38 | 0.50 | 47.1 | 0.26057 | 0.41983 | 0.32369 |
| 9 | 6.55 | 0.07 | 77.0 | 0.02029 | 0.02555 | 0.01319 |
| 7.08 | 6.73 | 0.13 | 23.9 | 0.03931 | 0.04602 | 0.02558 |
| 14 | 6.85 | 9.10 | 120 | 2.25119 | 2.00093 | 1.75480 |
| 7.05 | 7.09 | 0.03 | 14.6 | 0.00909 | 0.01252 | 0.00591 |
| 7.1 | 7.23 | 0.09 | 25.2 | 0.02724 | 0.03320 | 0.01771 |
| 7.04 | 7.29 | 0.02 | 27.2 | 0.00606 | 0.00873 | 0.00394 |
| 10.02 | 7.40 | 0.09 | 12.5 | 0.02724 | 0.03320 | 0.01771 |
| 6 | 7.50 | 0.29 | 1.4 | 0.00752 | 0.00153 | 0.00436 |
| 21 | 7.50 | 0.98 | 410 | 0.02496 | 0.00730 | 0.01474 |
| 7.06 | 7.62 | 0.04 | 15.3 | 0.00104 | 0.00012 | 0.00060 |
| 4 | 7.70 | 0.30 | 320 | 0.00778 | 0.00160 | 0.00451 |
| 10.01 | 7.70 | 0.06 | 14.3 | 0.00157 | 0.00020 | 0.00090 |
| 15 | 7.80 | 0.01 | 5.6 | 0.00034 | 0.00003 | 0.00020 |
| 19 | 8.05 | 0.12 | 1.7 | 0.00313 | 0.00049 | 0.00181 |
| 22 | 8.06 | 0.03 | 5.0 | 0.00078 | 0.00008 | 0.00045 |
| 1 | 8.27 | 0.02 | 219 | 0.00052 | 0.00005 | 0.00030 |
| 18 | 8.40 | 0.30 | 4.0 | 0.00778 | 0.00160 | 0.00451 |

*Data taken directly from (Wu et al., 2019)

Wu, P., Kainz, M. J., Bravo, A. G., Åkerblom, S., Sonesten, L., & Bishop, K. (2019). The importance of bioconcentration into the pelagic food web base for methylmercury biomagnification: A meta-analysis. *Science of The Total Environment*, *646*, 357-367.
